# Acutely calcified hematoma mimicking a displaced medial epicondyle fracture

**DOI:** 10.4103/0973-6042.57933

**Published:** 2009

**Authors:** Addie Majed, Joanna Thomas, Philip Ahrens

**Affiliations:** Department of Trauma and Orthopaedic Surgery, Royal Free Hospital, London, UK

**Keywords:** Supracondylar fracture, pin-track infection, calcified hematoma

## Abstract

We present an interesting and unusual case of an acutely calcified pin-site infection hematoma mimicking a displaced cartilaginous medial epicondyle, in a child with a Gartland type III fracture. The treatment of such pathology could be confusing and may interfere with the correct clinical decision-making process. To our knowledge, this is the first presentation of such a case.

## INTRODUCTION

Displaced supracondylar fractures of the humerus account for the second most common limb fractures in children and are associated with complications and poor results.[1] As a consequence, these injuries represent a management challenge and are best treated by accurate reduction.[2] Whilst Kirschner wires are versatile in fracture fixation within the pediatric group, complication rates are under-reported.[3]

We report a case of a calcified hematoma mimicking a displaced medial humeral epicondyle following a supracondylar fracture. The condition has not been previously documented in the medical literature.

## CASE REPORT

A 6-year-old boy, with no medical problems, presented to our Accident and Emergency Department following a fall onto his non-dominant outstretched left hand. Examination revealed a swollen painful elbow, with no skin lacerations and without distal neurovascular deficit. Radiographic evaluation confirmed a Gartland grade III supracondylar fracture of the left distal humerus with no other significant radiological findings.

The following morning, the patient underwent manipulation under anesthesia and stabilization of the fracture with two 1.6-mm Kirschner wires, positioned in a crossed fashion. There were no immediate postoperative complications, and the patient was discharged home 24 hours later in a split cast and sling immobilization.

**Figure 1:**
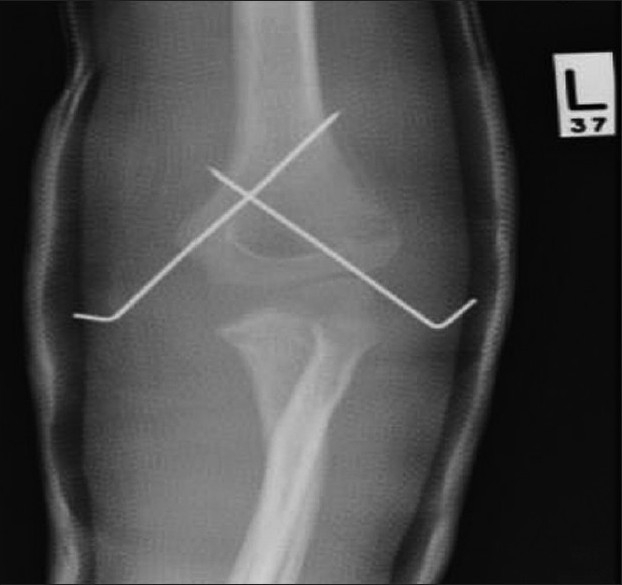
Anteroposterior radiograph demonstrating satisfactory fracture stabilisation

No complications were documented at routine follow-up 3 and 10 days postoperatively, and radiographs confirmed adequate fracture reduction and stabilization [Figure 1].

**Figure 2:**
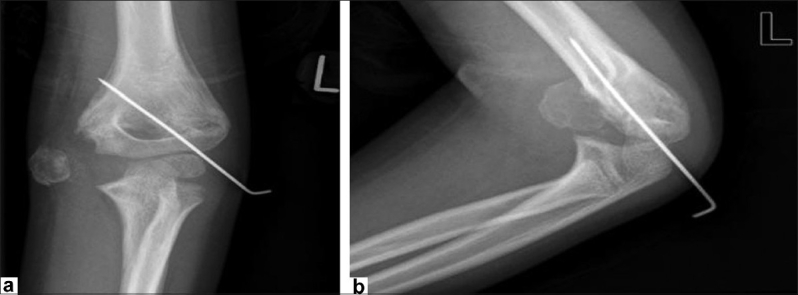
(a) Anteroposterior radiograph demonstrating a well circumscribed mass. (b) Lateral radiograph demonstrating a well circumscribed mass

Twenty-five days postoperatively, the patient presented to the Accident and Emergency Department with increased discomfort at the elbow. Examination revealed cellulitis surrounding, and a purulent discharge from, the medial pin site. As the medial Kirschner wire was loose and as there was clinical infection, the wire was removed. A microbiology swab was sent for culture and sensitivity, which later grew *Staphylococcus aureus*. The wound was cleaned and the patient was treated with intravenous third-generation cephalosporin antibiotics. At this time, radiographs demonstrated an apparent displacement of the medial epicondyle, which was enlarged, well circumscribed with a cortical outline and trabeculation [Figure 2a and b]. It was believed to represent calcification around the cartilaginous epicondyle secondary to displacement, hematoma and infection. A decision was made to treat any infection first, before considering re-operating. After 48 hours of intravenous antibiotics, the patient was discharged home on oral antibiotics. Review in the following week showed clinical resolution of the infection, a pain-free elbow and a good range of motion with no medial joint laxity. The decision was made to continue nonoperative management.

**Figure 3:**
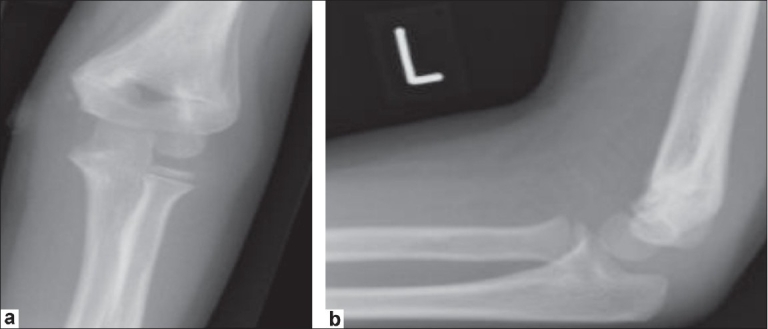
(a) Anteroposterior radiograph demonstrating the medial epicondyle in an anatomical position with union of the distal humerus. (b) Lateral radiograph demonstrating the medial epicondyle in an anatomical position with union of the distal humerus

Fifty-two days postoperatively, the patient was reviewed again in the outpatient department. Radiographs taken did not show a displaced medial epicondyle or nonunion and demonstrated normal soft tissue shadow and satisfactory alignment of the distal humerus [Figure 3a and b]. In fact, the medial epicondyle now revealed its ossification center and confirmed its normal anatomical position. The patient was clinically asymptomatic, he had a normal carrying angle and he regained full range of movement at the elbow.

In retrospect, a diagnosis of an acutely calcified hematoma with associated infection was made. Treating this patient's complication nonoperatively avoided an unnecessary surgical procedure and exposure to possible risks associated with it.

## DISCUSSION

Hematomas are typically fluid filled and subsequently develop a disconcertingly wide range of appearances depending on the degree of liquefaction and organization. Hematomas may be fluid filled, multiloculated or heterogeneous with solid and cystic elements. In this case, one must consider the rate of Kirschner wire-related complications, which include hematoma formation and infection. For example, in a series of 403 cases, Cheng *et al*. had 2 cases of superficial pin-tract infection,[5] whilst Botte *et al*. demonstrated a 7% rate of pin-tract infection.[6] Green described the factors associated with pin-tract infection, stressing motion at the pin-bone interface, necrotic bone or soft tissue around the pin and soft tissue tension where the pin exits the skin.[7]

Rajapakse *et al*. illustrated the difficulty of making the relatively rare diagnosis of a calcifying hematoma in the limbs by radiological means, and we have found that little has been written on calcifying hematoma.[8] These lesions can clinically and radiologically mimic aggressive soft tissue neoplasms, as outlined by Mentzel *et al*. in a series of 4 cases of calcified hematoma in the tensor fascia lata and perifascial tissue.[9] Additionally, myositis ossificans can also mimic these lesions.

To our knowledge, this is the first case of a calcified hematoma mimicking a displaced fracture fragment. Awareness of the possibility of this rare pathology and its radiological features may allow accurate diagnosis and prevent unnecessary surgery in an already challenging clinical situation.
